# The times have changed: molecular pathology is here to stay: A commentary on: Analytical performance of a PCR assay for the detection of KRAS mutations (codons 12/13 and 61) in formalin-fixed paraffin-embedded tissue samples of colorectal carcinoma, by Lee et al. In this issue

**DOI:** 10.1007/s00428-012-1197-z

**Published:** 2012-02-23

**Authors:** J. Han van Krieken, Gerald Hoefler

**Affiliations:** 1 Department of Pathology, Radboud University Nijmegen Medical Centre, PO Box 9101, 6500HB Nijmegen, The Netherlands; 2 Institute of Pathology, Medizinische Universitat Graz, Graz, Austria

Molecular pathology is now an integral part of diagnostic pathology. This has brought excitement and responsibilities to us as pathologists and molecular biologists in pathology—excitement, due to the many new possibilities we have for diagnosis, prediction of outcome, and response to therapy of disease, and responsibilities, since we need to perform the tests with high accuracy, which requires expertise. But is this different conventional histology and immunohistochemistry? At the time of Virchow, histopathology brought about enormous excitement, and as we are all aware, the responsibilities became rapidly clear. The first attempt to diagnose a tumor in 1882, on a biopsy of a Crown Prince, resulted in a failure, which led to many publications on this topic. Many of us remember the early days of immunohistochemistry, again enormous excitement, many publications (still ongoing) on its use, and obviously, great responsibilities since without immunohistochemistry, many diagnoses are impossible or, at the least, unreliable. But the pathologists looking down the microscope remained the final decision makers. And we all know that a good pathologist can make a correct diagnosis on a poor slide and/or a poor immunostained slide, but without knowledge and experience, even the best slide does not suffice. For molecular pathology, it is different.

For molecular tests (excluding fluorescent in situ hybridization, which is often regarded by pathologists as a bit fancier immunostain), the tissue is ground, DNA or RNA is extracted and processed, and bands, or colors or letters, appear on a screen, blot, or paper. These need to be interpreted in the context of the histological and immunohistochemical findings in order for a final conclusion to be drawn. This needs teamwork between a molecular biologist and pathologist, and both need to understand each other's trade. For pathologists, it is important to realize that there are factors that may influence the molecular results, factors such as fixation, tissue processing, and DNA extraction to name just a few. For molecular biologists, these issues are well known, but they also need to be informed about the exact diagnosis and the absolute and relative quantity of tumor cells and other potential test confounders. Not only that, both need to be aware of the test characteristics specific to the questions asked. That this is crucial can be learned from the KRAS story in colorectal cancer.

EGFR targeting agents have a modest effect on metastasized colorectal cancer. As it turned out, they are only effective on tumors with a wild-type KRAS gene, but in patients with a KRAS mutated tumor, the drug even has a negative effect. It is therefore mandatory that the test is performed with high accuracy. Therefore, the European Society of Pathology has initiated a European external quality assurance system, which is now in its fourth year [1-3]. Through this program, it has become clear not only that many different test systems can provide reliable results, but also that about 10–15% of the laboratories committed unacceptable mistakes (the good news, however, is that after feedback, these laboratories performed significantly better). It has also become clear that there are major differences in the pre-analytical phase between laboratories, that there are problems when, in a sample, the percentage of tumor cells to be tested is low, and that DNA extraction methods matter.

In this issue, Lee et al. describe a novel PCR-based method for KRAS testing and compare the results with the most commonly used test, Sanger sequencing [4]. This latter method is quite robust, but may show failures in case the sample contains a low percentage of tumor cells. The TaqMelt PCR assay—the cobas® KRAS Mutation Test—designed to detect 19 mutations in codons 12, 13, and 61 was shown to be superior in samples with a low percentage of tumor cells. The paper also discusses some of the issues which are relevant in molecular pathology of today and is therefore a valuable contribution to the literature, both for pathologists and for molecular biologists. It is, however, not the only commercial test system that provides highly accurate results in such samples. It is important that experiences like those from Lee et al. are published. The results of the ESP KRAS EQA system will be published as well. In this way, a large database with knowledge on methods of DNA extraction and test platforms will be built up. Such data will enable pathologists and molecular biologists to make an argued choice for test systems for any particular question. These choices are important, since each new method that is going to be used for molecular pathology needs in-house validation, prior to its application. This implies that one cannot change too often from one test to another, even though developments in methods of molecular testing evolve extremely fast.
